# Anti-Inflammatory Effects of Lipoxin A4 in *Salmonella* Typhimurium-Induced Enteritis in Wenchang Chickens

**DOI:** 10.3390/ani16030504

**Published:** 2026-02-05

**Authors:** Xiaoxiao Li, Hesi Ma, Jiankun Huang, Xuhua Ran, Xiaobo Wen

**Affiliations:** School of Tropical Agriculture and Forestry, Hainan University, Haikou 570228, China; 23220952000005@hainanu.edu.cn (X.L.); 18532399967@163.com (H.M.); jkhuang@hainanu.edu.cn (J.H.)

**Keywords:** *Salmonella Typhimurium*, Lipoxin A4, intestinal inflammation, anti-inflammatory, Wenchang chicken

## Abstract

*S. Typhimurium* infection has the capacity to elicit enteric inflammation and metabolic dysfunction among poultry. Prior research conducted by our laboratory observed an increase in LXA4 titers within the gut of Wenchang chickens following infection with *S. Typhimurium*. Based on this observation, the present study analyzed the changes in body weight, immune organ indices, the levels of intestinal inflammatory cytokines, as well as cyclooxygenase-2 (COX-2) expression in Wenchang chickens before and after infection. The findings indicated that *S. Typhimurium* infection led to reduced body weight and significantly decreased thymus and bursa indices. Furthermore, a significant elevation was observed in the transcript levels of pro-inflammatory mediators, including IL-1β, along with IL-6, and TNF-α, concurrently with an increase in the mRNA transcript levels of the enzyme COX-2. Treatment with LXA4 attenuated these alterations and effectively alleviated the inflammatory response. Additionally, an in vitro system was employed to validate the anti-inflammatory properties of LXA_4_ against *S. Typhimurium*-induced inflammation in chicken HD11 macrophages. The results demonstrated that LXA4 attenuated the transcript levels of IL-1β, as well as IL-6, TNF-α, and COX-2, at various intervals (2, 12, and 24 h), thereby alleviating inflammation elicited by *S. Typhimurium* challenge. We employed the LXA4 receptor antagonist Boc-2 to explore the ALX/FPR2 signaling axis and noted the successful neutralization of LXA4-mediated anti-inflammatory properties by this antagonist in *S. Typhimurium*–challenged HD11 macrophages. Collectively, these findings indicate that *S. Typhimurium* triggers pro-inflammatory reactions across both in vivo chicken models and in vitro HD11 macrophage systems, whereas LXA4 effectively mitigates this inflammatory process. This research establishes the conceptual underpinnings necessary to advance the design of therapeutic modalities aimed at counteracting *S. Typhimurium* challenges within poultry populations.

## Simple Summary

*Salmonella* Typhimurium (*S. Typhimurium*) is a widely distributed zoonotic enteric pathogen and the most frequently reported serovar associated with *Salmonella* infections. It is also one of the principal causative agents of *salmonellosis* in poultry. Infection with *S. Typhimurium* typically results in reduced growth rate, impaired feed conversion efficiency, and increased mortality in young chicks. Wenchang chicken, an indigenous and economically valuable meat-type breed from Hainan Province, is integral to elevating poultry product quality while fostering the progression of specialized poultry production systems. However, this breed is particularly susceptible to *S. Typhimurium* infection, which often leads to severe intestinal inflammation and diarrhea, thereby posing substantial risks to health status and growth performance. Serving as an archetypal specialized pro-resolving mediator (SPM), Lipoxin A4 (LXA4) modulates immune cell activation and facilitates the restoration of tissue homeostasis. Given its central role in actively orchestrating the resolution of inflammation, increasing attention has been directed toward its regulatory functions in intestinal inflammatory responses. Based on its well-documented pro-resolving activity, the current study seeks to determine the protective function of LXA4 during the alleviation of *S. Typhimurium*-induced intestinal inflammation in Wenchang chickens, thereby providing a foundation for establishing new anti-inflammatory strategies and contributing to the sustainable and healthy progression of the poultry sector.

## 1. Introduction

*S. Typhimurium* is a significant Gram-negative enteric pathogen widely distributed in poultry, mammals, and the environment, serving as a primary causative agent of avian infections [1]. Infection with *S. Typhimurium* can induce intestinal inflammation, diarrhea, weight loss, and systemic diseases in poultry, potentially leading to sepsis or mortality, thereby causing substantial economic losses in the poultry industry and posing public health risks due to its zoonotic potential [2]. The pathogen infiltrates the enteric epithelium by means of its type III secretion system (T3SS), injecting effector proteins that trigger inflammatory cascades [3]. In poultry, this often results in acute enteritis accompanied by excessive activation of neutrophils and macrophages, compromising the intestinal barrier and causing histopathological changes. While host immune responses aim to clear the pathogen, an excessive liberation of pro-inflammatory mediators, exemplified by IL-1β and TNF-α, can aggravate tissue damage [4,5,6]. Furthermore, increasing antibiotic resistance in *S. Typhimurium* complicates treatment and control efforts [7]. Given the unique aspects of avian immunity, such as the absence of lymph nodes and distinct macrophage functions, pathogen-host interactions differ from mammalian models, highlighting the need for targeted strategies to modulate inflammation and develop novel anti-infective approaches [8].

Arachidonic acid (AA) constitutes a major ω-6 polyunsaturated fatty acid that is primarily localized and esterified within membrane phospholipids [9]. Upon cellular stimulation, phospholipase A2 (PLA_2_) liberates unbound arachidonic acid (AA), which undergoes biotransformation through three primary enzymatic routes—namely the COX, LOX, and CYP450 cascades—to generate bioactive lipid mediators [10].

The COX metabolic system mediates the formation of prostanoids, specifically prostaglandins (PGs) and thromboxanes (TXs), which are integrally involved in inflammation, vasodilation, and platelet aggregation. Simultaneously, the lipoxygenase (LOX) enzymatic branch, which is regulated by the catalytic activities of 5-LOX, 12-LOX, and 15-LOX isoforms, yields a diverse array of metabolites including leukotrienes (LTs), hydroxyeicosatetraenoic acids (HETEs), and lipoxins (LXs). Within this network, LTs primarily promote the recruitment of inflammatory cells, whereas LXs possess potent anti-inflammatory properties and facilitate pro-resolving activities [11]. The CYP450 pathway yields epoxyeicosatrienoic acids (EETs) and 20-HETE, regulating vascular tone and cytoprotection. These mediators dynamically modulate immune responses, cell signaling, and tissue repair, with imbalances linked to inflammatory diseases, making them promising therapeutic targets [12]. In intestinal inflammation, AA metabolites play dual roles: pro-inflammatory (e.g., LTB_4_ enhancing neutrophil infiltration) and anti-inflammatory (e.g., LXA4 promoting resolution) [13,14,15,16,17].

As an endogenous lipid mediator with anti-inflammatory activity, LXA4 is derived from AA via the LOX pathway and serves as a SPM [18]. It regulates neutrophil, macrophage, and lymphocyte functions, suppressing pro-inflammatory signals (e.g., NF-κB, MAPK) and promoting apoptotic cell clearance, thereby facilitating resolution in acute and chronic inflammation [19,20]. Biosynthesis primarily involves 15-LOX and 5-LOX collaboration, often through transcellular mechanisms, with aspirin-triggered lipoxins (ATLs) offering enhanced stability. LXA4 signals via the ALX/FPR2 receptor [21], inhibiting NF-κB and modulating MAPK pathways to attenuate the production of pro-inflammatory cytokines such as TNF-α and IL-6, while interacting with other mediators like LTB_4_. In immune cells, LXA4 inhibits neutrophil chemotaxis and NET formation, enhances macrophage efferocytosis and M2 polarization, and modulates dendritic and T cell responses [22]. Recent studies highlight its protective roles in models of pneumonia, colitis, and arthritis, with clinical correlations in asthma, Systemic Lupus Erythematosus (SLE), and Rheumatoid Arthritis (RA) [23]. Recent studies have developed metabolically more stable analogs of LXA4 through chemical synthesis [24]. These analogs, acting as partial agonists of the ALX/FPR2 receptor, more effectively suppress the NF-κB signaling cascade and a broad array of pro-inflammatory mediators. These advances provide important theoretical and experimental foundations for the development of novel anti-inflammatory strategies targeting the LXA4 signaling pathway [25].

Despite significant advances in understanding the pathogenesis of *S. Typhimurium* infection and arachidonic acid (AA) metabolism, the role of lipoxin A4 (LXA4) in modulating intestinal inflammation in poultry and macrophage responses to infection remains incompletely understood. Therefore, in this study, a dual methodology encompassing both in vivo and in vitro experiments was employed to further elucidate the anti-inflammatory effects and underlying mechanisms of LXA4 in enteric inflammation elicited by *S. Typhimurium* in poultry. (1) The alterations in IL-1β, along with IL-6, TNF-α, and COX-2, within the cecal tissues were assessed utilizing quantitative real-time PCR (qRT-PCR) techniques; (2) the effects of LXA4 on immune organ indices in Wenchang chickens with enteritis induced by *S. Typhimurium* infection were evaluated; (3) the cytotoxicity and bactericidal potential of LXA4 in HD11 macrophages were assessed; (4) the influence of LXA4 regarding the survival of *S. Typhimurium*-infected chicken HD11 macrophages was evaluated via the CCK-8 colorimetric assay; (5) the transcript levels of IL-1β as well as IL-6, TNF-α, and COX-2 were quantified via qRT-PCR in chicken HD11 cells at 2, 12, and 24 h following infection; (6) the involvement of the ALX/FPR2 signaling pathway was investigated using the ALX/FPR2 antagonist Boc-2. These findings may inform novel strategies for controlling *S. Typhimurium* in poultry.

## 2. Materials and Methods

### 2.1. Ethics Statement

The protocol for all animal experiments was formally sanctioned by the Ethics Committee of Hainan University (Haikou, China), under the reference number HNUAUCC-2023-00080. Furthermore, the authors confirm that no animal species utilized in this research are classified as endangered or under legal protection.

### 2.2. Bacterial Strain

Following procurement from Guangdong Huankai Microbial Science and Technology Co., Ltd. (Guangzhou, China), *S. Typhimurium* ATCC 14028 was resuscitated from cryogenic glycerol stocks (−80 °C). The subculturing process involved a 12 h duration within LB broth at 37 °C, with the flasks placed on a rotary shaker at 200 rpm to facilitate adequate aeration and maximize cell viability prior to inoculation.

### 2.3. LXA4 and Boc-FLFLF (Boc-2)

High-purity Lipoxin A4 (LXA4, ≥95%) was procured from Cayman Chemical (Ann Arbor, MI, USA). For in vivo gavage, the compound was reconstituted to the required potency using ice-cold PBS. To prepare an in vitro stock solution (10,000 nM), 35.25 µL of LXA4 was meticulously measured and dissolved in 1 mL of chilled PBS, followed by sterilization through a 0.22 µm membrane filter. This stock was utilized within 24 h of preparation and maintained at −80 °C during the interim.

Similarly, Boc-2—a selective antagonist targeting the ALX/FPR2 receptor—was obtained from Cayman Chemical (≥95% purity). For experimental assays, a 1 mM stock was established via the dissolution of a 1 mg quantity of Boc-2 within a 1.272 mL volume of dimethyl sulfoxide (DMSO). Following 0.22 µm syringe filtration to ensure sterility, the solution was aliquoted for single use and stored at −20 °C to avoid detrimental freeze–thaw cycles. Final working solutions were formulated by diluting the stock with culture medium immediately preceding the experiments.

### 2.4. Experimental Animals and Study Groups

A total of thirty healthy, day-old Wenchang chickens underwent randomized allocation into five distinct cohorts (*n* = 6 per group), with initial body weight homogeneity maintained across all groups throughout the seven-day experimental trial. To minimize external interference, both the control and treatment groups were raised without vaccination or the addition of anticoccidial drugs. Before the formal experiment, all Wenchang cockerels underwent fecal screening via sterile cloacal swabbing to rule out *S. Typhimurium* colonization. Culture on SS agar confirmed the absence of infection, allowing for the subsequent randomization of the pathogen-free chicks into five groups.

(1) Healthy control group (CON): chickens were fed normally without infection.
(2) *S. Typhimurium* infection group (ST): Each chicken was subjected to daily oral gavage with 0.3 mL of the bacterial inoculum (1 × 10^6^ CFU/mL) [26] over three successive days.
(3) *S. Typhimurium* + 5 µg/kg LXA4 treatment group (ST + 5 µg/kg LXA4): after infection as described above, chickens were orally administered LXA4 at 5 µg/kg once daily for three consecutive days.
(4) *S. Typhimurium* + 10 µg/kg LXA4 treatment group (ST + 10 µg/kg LXA4): after infection, chickens received LXA4 at 10 µg/kg once daily for three days.
(5) *S. Typhimurium* + 20 µg/kg LXA4 treatment group (ST + 20 µg/kg LXA4): after infection, chickens received LXA4 at 20 µg/kg once daily for three days.

We designated the day of arrival for the one-day-old Wenchang chickens as experimental day 1. Inoculation with *S. Typhimurium* was performed via oral gavage on the first three consecutive days. On days 2 and 3, fecal material was harvested with sterile swabs, resuspended in phosphate-buffered saline (PBS), and 100 μL of the homogenate was cultured on SS agar. Following a 24 h incubation period at 37 °C, bacterial colonization was assessed. The therapeutic stage was triggered only after confirming that all Wenchang chickens had been successfully infected with *S. Typhimurium*. All chickens were maintained with ad libitum intake of a standardized diet coupled with fresh water for the whole duration of the trial. The ambient temperature in the housing facility was strictly regulated and maintained at 32–35 °C using supplemental heaters to ensure an optimal thermal environment for the chickens. The room was disinfected 1 to 2 times a day. Both control and experimental cohorts were maintained under identical conditions.

### 2.5. Verification of the Infection Model

To validate successful colonization, fecal samples were obtained 12 h post-inoculation and streaked onto SS agar plates. Following an 18–24 h cultivation period at 37 °C, the plates underwent inspection to identify distinctive *Salmonella* spp. colonies—characterized by their colorless appearance with central black pigmentation—thereby confirming the successful induction of the experimental infection model in Wenchang chickens.

### 2.6. Sample Collection

On day 7 of the experiment, all Wenchang cockerels were humanely euthanized via cervical dislocation. The immune organs, including the thymus, spleen, and bursa of Fabricius, were aseptically harvested on ice and weighed for documentation. Subsequently, representative segments of the mid-ceca (approximately 1–2 cm in length) were excised using sterile scissors. The cecal contents were gently removed, and the remaining cecal wall tissues were thoroughly rinsed with ice-cold sterile phosphate-buffered saline (PBS). These tissue samples were instantly cryopreserved within liquid nitrogen for 8 h prior to their relocation to a −80 °C freezer for subsequent analyses.

### 2.7. Clinical Severity Grading

The clinical status of the respective Wenchang chicken groups was assessed and scored daily. The specific metrics for these evaluations are detailed in Table 1.

### 2.8. Real-Time Fluorescence Quantitative PCR

RNA Isolation and RT-qPCR Analysis: HD11 macrophages were seeded into 6-well plates at a density of 1 × 10^5^ cells per well. After pre-treatment with LXA4 or Boc-2 and subsequent *S. Typhimurium* challenge (MOI = 20), cellular samples were collected at 2, 12, and 24 h after infection. Before being lysed with TRIzol reagent, the cell monolayers were washed three times using PBS.

For the isolation of total RNA from cecal samples, the RaPure Total RNA Mini Kit was employed, with a spectrophotometer used to assess RNA purity and concentration. Reverse transcription was executed using ABScript Neo RT Master Mix (ABclonal Technology, Wuhan, China) containing gDNA remover to synthesize cDNA. The qPCR assays were conducted in a 10 µL volume utilizing SYBR Green. The 2^−△△Ct^ method was applied to quantify the relative expression of target genes, utilizing β-actin as the internal reference gene for normalization. Table 2 provides a comprehensive list of the primer sequences used.

### 2.9. LXA4 In Vitro Inhibitory Concentration

To resuscitate *S. Typhimurium*, the glycerol-preserved stocks were streaked onto SS agar and incubated overnight at a constant 37 °C to ensure optimal bacterial recovery. Bacterial populations in the mid-exponential phase (OD600 within 0.6–0.8) were obtained by inoculating LB broth with single colonies at a 1:100 ratio for 8 h. LXA4 underwent sequential dilution within LB broth to establish a concentration gradient (0, 7.81, 15.6, 31.3, 62.5, 125, 250, 500, and 1000 nM). The proliferation kinetics of *S. Typhimurium* were determined at 2, 4, 6, and 8 h intervals during incubation at 37 °C with constant orbital agitation (200 rpm). Viable bacteria were enumerated using the standard spread-plate method, with results expressed as CFU.

### 2.10. Assessment of HD11 Cell Viability Under Varying LXA4 and Boc-2 Concentrations via CCK-8 Measurement

HD11 macrophages (Shanghai Yage Biotechnology Co., Ltd., Shanghai, China) were seeded in 96-well microplates at an initial count of 1 × 10^4^ cells in each respective well (100 µL per well). Following a period to allow for confluent attachment, we discarded the supernatant and performed three successive rinses of the cell monolayers using PBS. To determine cytotoxicity, the cells were subjected to a concentration gradient of LXA4 or Boc-2 (diluted in DMEM), specifically 0, 7.81, 15.6, 31.3, 62.5, 125, 250, 500, and 1000 nM. Incubation lasted for intervals of 4, 16, or 24 h, with each specific condition conducted in triplicate. At the end of each exposure time, the CCK-8 reagent was added to each well for a further 2 h incubation. Subsequently, the optical density (OD) at a wavelength of 490 nm was quantified using a microplate reader. Our experimental setup included a blank control (medium without cells) as well as a negative control (cells without drug treatment). The percentage of living cells was determined according to the formula described below: Cell viability (%) = [(OD490 of experimental group − OD490 of blank group)/(OD490 of negative control group − OD490 of blank group)] × 100

### 2.11. Statistical Analysis

Preliminary data management was conducted in Excel 2019. Data normality within each group and homogeneity of variances among groups were assessed using the Shapiro–Wilk test. For parameters exhibiting a normal distribution and homogeneous variances (clinical symptom scores, body weight, thymus and bursa of Fabricius indices, cytotoxicity of different concentrations of LXA4 after 4 h and 16 h, transcriptional levels of animal inflammatory factors IL-1β, IL-6, and COX-2, effect of LXA4 and Boc-2 on *S. Typhimurium*-infected HD11 macrophages, and mRNA expression levels of inflammatory cytokines at different time points), inter-group differences were evaluated via one-way ANOVA (or two-way ANOVA where applicable), subsequently employing Duncan’s multiple range test for post hoc comparisons.

For datasets failing to meet normality assumptions (including the spleen index, TNF-α mRNA expression levels in inflammatory animal models, and the cytotoxicity of LXA4 at varying concentrations after 24 h), the Kruskal–Wallis test was first employed to evaluate global significance among all experimental groups. If a significant difference was observed (*p* < 0.05), post hoc pairwise comparisons were performed using the Mann–Whitney U test to identify specific differences between groups. All statistical calculations and visualizations were performed using GraphPad Prism 9.0, and data are expressed as mean ± SEM for consistency. The criterion for statistical significance was established at *p* < 0.05.

## 3. Results

### 3.1. Clinical Manifestations and Weight Dynamics in Wenchang Chickens Subjected to S. Typhimurium Infection

**Figure 1:**
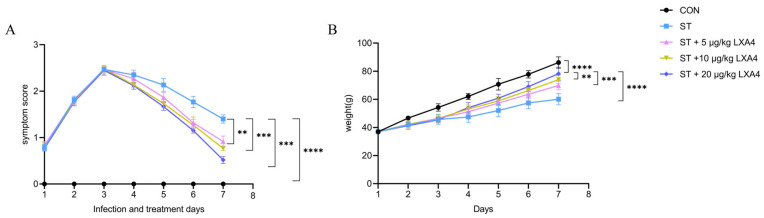
(**A**) Clinical symptom scores of Wenchang chickens post *S. Typhimurium* infection and LXA4 treatment. Higher scores indicate more severe symptoms. (**B**) Body weight changes in Wenchang chickens under different LXA4 treatment doses following *S. Typhimurium* infection. ** *p* < 0.01, *** *p* < 0.001, **** *p* < 0.0001 (significant).

Wenchang chickens infected with *S. Typhimurium* exhibited lethargy, reduced activity, and depressed behavior at 48 h post-infection. Fecal cultures on SS agar plates confirmed successful infection. At 48 h post-infection, the average bacterial shedding in the infected group reached 3.5 × 10^4^ CFU/g of feces, which is consistent with the early colonization levels of *S. Typhimurium* in chicken models reported in previous studies [27]. The data for both clinical symptom scores and body weight followed a normal distribution (Shapiro–Wilk test, *p* > 0.05). Clinical monitoring revealed that the ST group exhibited severe symptoms, with scores remaining elevated until the end of the experiment (Day 7 score: 1.40 ± 0.09, *p* < 0.0001). However, LXA4 treatment significantly alleviated these symptoms in a dose-dependent manner. By day 7, the 20 µg/kg LXA4 group showed the most pronounced recovery (score: 0.52 ± 0.08, *p* < 0.001), followed by the 10 µg/kg group (0.77 ± 0.05, *p* < 0.001) and the 5 µg/kg group (0.92 ± 0.12, *p* < 0.01) (Figure 1A).

Consistently, body weight monitoring revealed that *S. Typhimurium* infection caused significant weight loss. By day 7, the body weight of the ST group was significantly lower than that of the control group (60.07 ± 4.03 g vs. 86.22 ± 4.01 g, *p* < 0.0001). LXA4 treatment effectively mitigated this weight loss. The 20 µg/kg group maintained the highest body weight (78.21 ± 4.60 g, *p* < 0.0001), which was significantly higher than that of the model group. The 10 µg/kg and 5 µg/kg groups also showed improved body weights of 73.97 ± 2.94 g (*p* < 0.01) and 69.82 ± 3.04 g (*p* < 0.001), respectively (Figure 1B). These findings indicate that LXA4 effectively alleviates clinical symptoms and prevents post-infection weight loss in Wenchang chickens.

### 3.2. Impact of LXA4 on the Immune Organ Indices of Wenchang Chickens Subjected to S. Typhimurium Infection

**Figure 2:**
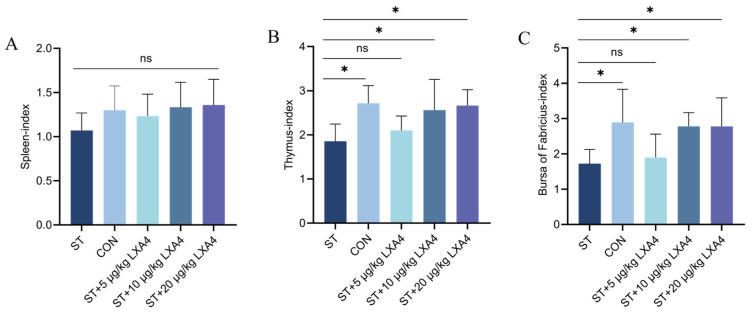
(**A**) Effect of LXA4 on the immune organ indices of Wenchang chickens infected with *S. Typhimurium*. (**A**) Spleen index; (**B**) thymus index; (**C**) bursa of Fabricius index. ns, *p* > 0.05 (no significance); * *p* < 0.05 (significant).

The influence of LXA4 on immune organs within the Wenchang chicken model challenged with *S. Typhimurium* was evaluated by calculating the organ-to-body weight ratios of the spleen, thymus, and bursa of Fabricius (Figure 2).

The spleen indices were analyzed using the Kruskal–Wallis test, which revealed no significant global differences among the experimental groups (H = 4.447, *p* = 0.3488). Consequently, although Mann–Whitney U tests were performed for pairwise comparisons, no statistically significant differences were observed between any two groups (*p* > 0.05). The spleen index of Wenchang chickens showed no significant differences among the ST group and the CON group (*p* = 0.379), the ST + 5 µg/kg LXA4 group (*p* = 0.662), the ST + 10 µg/kg LXA4 group (*p* = 0.265), or the ST + 20 µg/kg LXA4 group (*p* = 0.197) (Figure 2A; *p* > 0.05). These observations align with the finding that *S. Typhimurium*-induced inflammation is largely confined to the intestinal barrier and hepatic portal system, without eliciting a pronounced systemic immune response or splenic follicular hyperplasia. As a result, changes in spleen index were not statistically significant.

In contrast, the thymus index displayed clear alterations (Figure 2B). The data for the thymus index and the Bursa of Fabricius index all followed a normal distribution (Shapiro–Wilk test, *p* > 0.05). Relative to the CON group, the ST group exhibited a significant reduction (*p* = 0.011; *p* < 0.05). Although a slight improvement was observed in the ST + 5 µg/kg LXA4 group (*p* = 0.757), the difference was not significant (*p* > 0.05). Notably, supplementation with 10 or 20 µg/kg LXA4 resulted in marked recovery of the thymus index (*p* = 0.041 and *p* = 0.0172, respectively; *p* < 0.05), indicating a dose-responsive protective effect of LXA4.

Similarly, the bursa of Fabricius index exhibited a marked decline within the ST cohort in comparison to the CON group (*p* = 0.021; *p* < 0.05); (Figure 2C). Treatment with 5 µg/kg LXA4 produced only minimal, non-significant improvement (*p* = 0.976; *p* > 0.05), whereas both the 10 and 20 µg/kg LXA4 groups showed significantly better restoration of the bursal index (*p* = 0.041 and *p* = 0.040, respectively; *p* < 0.05). These experimental results collectively demonstrate that LXA4 ameliorates immune organ damage caused by *S. Typhimurium* infection, particularly at higher doses.

### 3.3. Impact of LXA4 on the Expression of Intestinal Pro-Inflammatory Mediators in Wenchang Chickens Subjected to S. Typhimurium Infection

The mRNA expression levels of inflammatory mediators were evaluated based on their data distribution. For TNF-α, which exhibited a non-normal distribution, the Kruskal–Wallis test revealed significant global variations among the treatment groups (H = 18.30, *p* = 0.0011). In contrast, the mRNA levels of IL-1β, IL-6, and COX-2 followed a normal distribution (Shapiro–Wilk test, *p* > 0.05) and were analyzed using one-way ANOVA.

**Figure 3:**
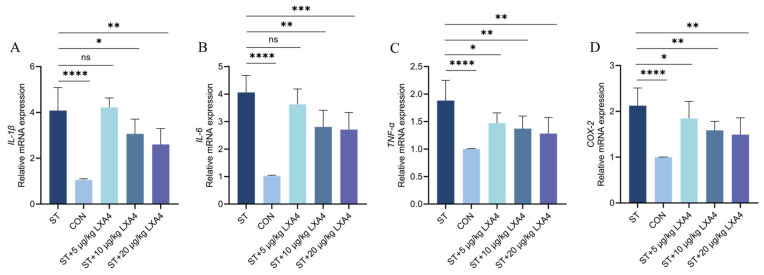
Effect of LXA4 on the transcript levels of inflammatory factors in the cecum of Wenchang chickens. Data represent individual biological replicates (*n* = 6 per group); no sample pooling was performed. (**A**) IL-1β; (**B**) IL-6; (**C**) TNF-α; (**D**) COX-2 enzyme. ns, *p* > 0.05 (no significance); * *p* < 0.05, ** *p* < 0.01, *** *p* < 0.001, **** *p* < 0.0001 (significant).

In comparison with the CON group, the relative mRNA expression levels of pro-inflammatory factors, including IL-1β, IL-6, TNF-α, and COX-2, were detected in the ST group (*p* < 0.0001), signifying the development of severe cecal inflammation following *S. Typhimurium* challenge. Regarding the treatment groups, the 5 µg/kg LXA4 dose did not produce statistically significant changes in IL-1β and IL-6 mRNA transcriptional levels (*p* > 0.05; Figure 3A,B); however, a notable decrease in the transcript levels of TNF-α and COX-2 was observed (*p* < 0.05; Figure 3C,D). Furthermore, compared to the ST group, both 10 and 20 µg/kg LXA4 treatments successfully downregulated the relative mRNA transcripts for all measured mediators (*p* < 0.05).

Collectively, these data demonstrate that LXA4 exerts a protective effect by potently suppressing cecal inflammatory signaling, thereby mitigating enteric inflammation in *S. Typhimurium*-infected Wenchang chickens. Specifically, while *S. Typhimurium* infection triggers a surge in intestinal cytokine expression, the administration of LXA4 at doses of 10 μg/kg and 20 μg/kg effectively suppressed these inflammatory markers, promoting the resolution of enteric inflammatory responses in Wenchang chickens.

### 3.4. Evaluation of the Direct Antibacterial Activity of LXA4 Against S. Typhimurium In Vitro

**Figure 4:**
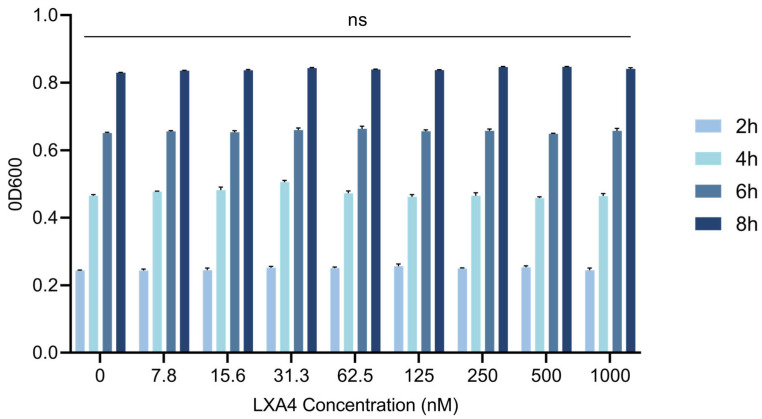
Effects of different concentrations of LXA4 on *S. Typhimurium* growth. ns, *p* > 0.05 (no significance).

To assess the potential antibacterial activity of LXA4 against *S. Typhimurium*, bacteria were exposed to a series of concentrations (0–1000 nM) and evaluated at 2, 6, and 8 h post-treatment. No significant inhibition of bacterial growth was observed within 8 h at any tested concentration (Figure 4), indicating that LXA4 does not exert direct bactericidal effects against *S. Typhimurium*.

These results suggest that LXA4 primarily mediates its protective effects by modulating host immune responses rather than directly suppressing bacterial proliferation. Specifically, LXA4 enhances macrophage clearance of *S. Typhimurium*, mitigates inflammatory damage, and promotes cellular resilience during infection.

### 3.5. Effect of Different Concentrations of LXA4 on HD11 Macrophage Viability

To evaluate the potential cytotoxicity of LXA4 on HD11 chicken macrophages, cellular survival was assessed using the CCK-8 assay. Data at 24 h exhibited a non-normal distribution and were analyzed using the Kruskal–Wallis test (H = 14.79, *p* = 0.0634) followed by Mann–Whitney U tests for pairwise comparisons, whereas data at 4 h and 16 h followed a normal distribution (Shapiro–Wilk test, *p* > 0.05) and were evaluated via one-way ANOVA.

**Figure 5:**
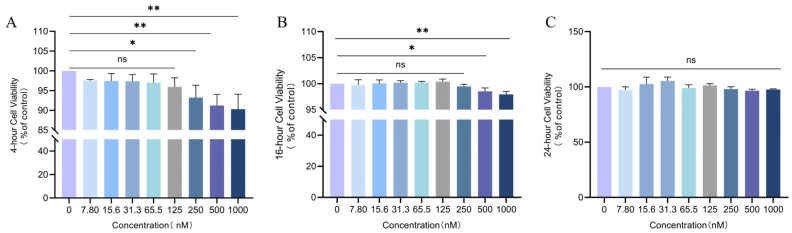
Cytotoxicity of different concentrations of LXA4 after 4 h (**A**), 16 h (**B**) and 24 h (**C**) exposure. ns, *p* > 0.05 (no significance); * *p* < 0.05, ** *p* < 0.01 (significant).

Following 24 h of treatment, LXA4 at 1000 nM exhibited no marked impact on cellular survival versus the control (*p* > 0.05), indicating no cytotoxicity (Figure 5C). At 16 h, concentrations ≥500 nM significantly compromised cellular viability (*p* < 0.05), suggesting cytotoxic effects (Figure 5B). At 4 h, concentrations ≥250 nM also significantly reduced viability (*p* < 0.05) (Figure 5A).

Accordingly, subsequent experiments investigating the influence of LXA4 on *S. Typhimurium*-infected HD11 cells were performed using concentrations below 250 nM (50, 100, and 200 nM) and shorter treatment durations (3, 6, and 12 h) to avoid cytotoxicity.

### 3.6. Effect of LXA4 on S. Typhimurium-Infected HD11 Macrophages

**Figure 6:**
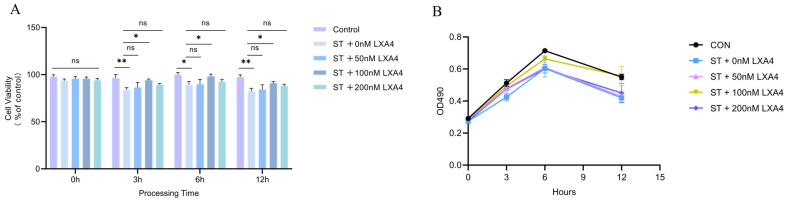
Effects of LXA4 on the viability of HD11 macrophages infected with *S. Typhimurium*. Values denote mean ± SEM. (**A**) Cell viability of HD11 macrophages measured by CCK-8 assay at 0, 3, 6, and 12 h post-infection; (**B**) Growth curves of HD11 cells under different treatment conditions monitored by OD490 absorbance. ns, *p* > 0.05 (no significance); * *p* < 0.05, ** *p* < 0.01 (significant).

To investigate the cytoprotective potential of LXA4, HD11 macrophages were subjected to *S. Typhimurium* infection (ST group), which resulted in a significant, time-dependent reduction in cell viability compared to uninfected controls (*p* < 0.05). While treatment with 50 nM and 200 nM LXA4 failed to significantly alleviate infection-induced cytotoxicity (*p* > 0.05), the administration of 100 nM LXA4 demonstrated a protective effect on cellular survival at 3 h, 6 h, and 12 h (*p* < 0.05; Figure 6A).

Consequently, these data identify 100 nM as the optimal concentration for mitigating *S. Typhimurium* infection in HD11 cells (Figure 6B), and this dosage was selected for subsequent analyses regarding the regulatory impact of LXA4 on inflammatory transcript levels.

### 3.7. Effect of Different Concentrations of the ALX/FPR2 Antagonist Boc-2 on HD11 Macrophage Viability

**Figure 7:**
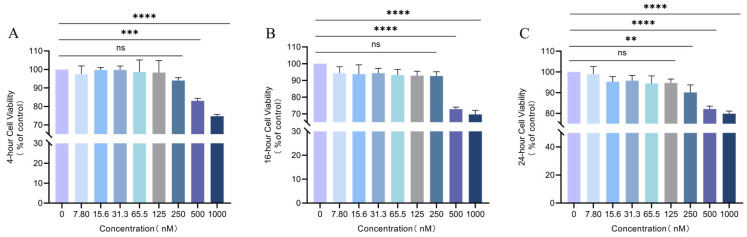
Cytotoxicity of different Boc-2 concentrations after 4 h (**A**), 16 h (**B**) and 24 h (**C**) exposure. ns, *p* > 0.05 (no significance); ** *p* < 0.01, *** *p* < 0.001, **** *p* < 0.0001 (significant).

Similarly, CCK-8 assays were performed to assess the effects of the ALX/FPR2 antagonist Boc-2 on HD11 cell viability. At 24 and 16 h, Boc-2 at concentrations ≤ 250 nM exhibited no marked impact on cellular survival relative to the control (*p* > 0.05), indicating no cytotoxicity (Figure 7B,C). However, at 4 h, concentrations of 500 nM and 1000 nM significantly reduced cell viability (*p* < 0.0001) (Figure 7A).

Based on these findings, subsequent experiments investigating the effect of Boc-2 on *S. Typhimurium*-infected HD11 cells were conducted using concentrations below 500 nM (100, 200, and 400 nM) and exposure times of 3, 6, and 12 h to avoid cytotoxic effects.

### 3.8. Effect of the ALX/FPR2 Antagonist Boc-2 on S. Typhimurium-Infected HD11 Macrophages

**Figure 8:**
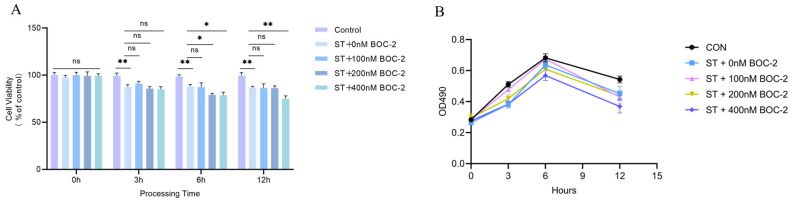
Effect of Boc-2 on the viability of *S. Typhimurium*-infected HD11 macrophages. (**A**) Cell viability of HD11 macrophages measured by CCK-8 assay at 0, 3, 6, and 12 h post-infection; (**B**) Growth curves of HD11 cells under different treatment conditions monitored by OD490 absorbance. ns, *p* > 0.05 (no significance); * *p* < 0.05, ** *p* < 0.01 (significant).

To evaluate the impact of the ALX/FPR2 antagonist Boc-2 on *S. Typhimurium*-infected HD11 macrophages, cell viability was assessed to determine an appropriate concentration for subsequent gene expression analysis. The results showed that cell viability gradually declined over time following infection with *S. Typhimurium*. Compared to the *S. Typhimurium*-infected control group (ST + 0 nM Boc-2 group), treatment with 400 nM Boc-2 significantly reduced cell viability at 6 h and 12 h (*p* < 0.05), and the 200 nM concentration also showed significant cytotoxicity at 6 h (*p* < 0.05). In contrast, 100 nM Boc-2 demonstrated no significant difference in cell viability compared to the infected control group (*p* > 0.05), indicating that this dosage is non-toxic to *S. Typhimurium*-infected cells (Figure 8A).

Based on these findings, a concentration of 100 nM Boc-2 was selected for subsequent experiments to evaluate its effect on inflammation-related gene expression (Figure 8B).

### 3.9. Effect of LXA4 on COX-2 mRNA Expression in S. Typhimurium-Infected HD11 Macrophages

The normality of the data was confirmed using the Shapiro–Wilk test (*p* > 0.05). The results demonstrated that treatment of normal HD11 macrophages with 100 nM LXA4 (CON + LXA4 group) did not induce significant changes regarding transcript expression of COX-2 relative to the control group (CON group) across intervals of 2, 12, and 24 h (*p* > 0.05), indicating that LXA4 treatment did not significantly alter COX-2 mRNA levels in normal HD11 cells without inflammatory stimulation.

**Figure 9:**
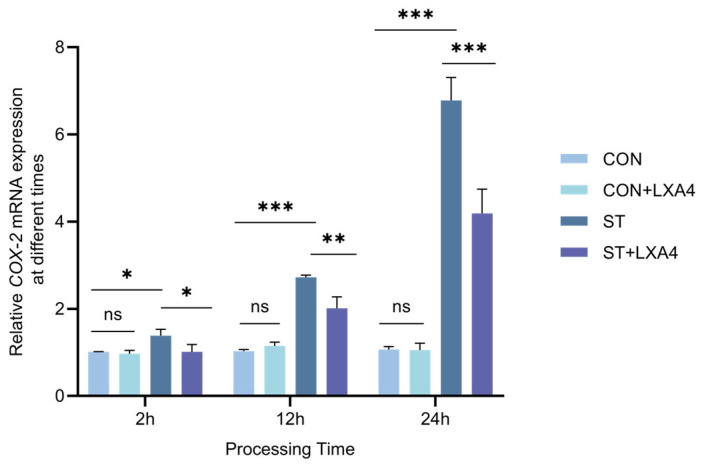
Effect of LXA4 on COX-2 mRNA expression in HD11 cells after 2, 12 and 24 h infection with *S. Typhimurium*. ns, *p* > 0.05 (no significance); * *p* < 0.05, ** *p* < 0.01, *** *p* < 0.001 (significant).

At 2 and 12 h post-infection with *S. Typhimurium*, HD11 macrophages exhibited a slight increase in COX-2 mRNA transcriptional levels relative to the normal group (*p* < 0.005), an elevation that was notably attenuated following LXA4 treatment. At 24 h post-infection, HD11 macrophages showed a significant elevation in COX-2 mRNA expression compared with the CON group (*p* < 0.001), which markedly declined after LXA4 treatment, as illustrated in Figure 9.

Infection with *S. Typhimurium* alone (ST group) precipitated a substantial induction of COX-2 at the transcriptional level (*p* < 0.001). Notably, treatment with LXA4 in infected cells (ST + LXA4 group) significantly reduced COX-2 expression at 2, 12, and 24 h following infection relative to the ST group (*p* < 0.005). Collectively, these observations demonstrate that LXA4 effectively mitigates *S. Typhimurium*-induced COX-2 overexpression in HD11 macrophages.

### 3.10. Effect of LXA4 on Inflammatory Cytokine Expression in S. Typhimurium-Infected HD11 Macrophages

**Figure 10:**
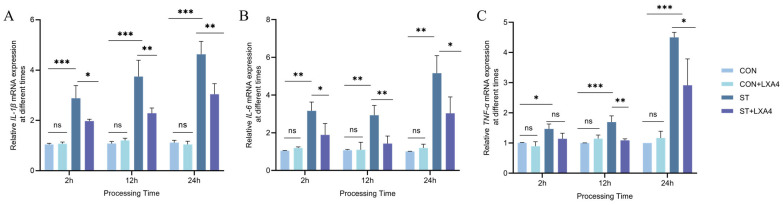
Effect of LXA4 on IL-1β (**A**), IL-6 (**B**), TNF-α (**C**) mRNA expression in HD11 macrophages after 2, 12 and 24 h infection with *S. Typhimurium*. ns, *p* > 0.05 (no significance); * *p* < 0.05, ** *p* < 0.01, *** *p* < 0.001 (significant).

To assess the anti-inflammatory potential of LXA4 on *S. Typhimurium*-infected HD11 macrophages, the relative mRNA expression levels of IL-1β (Figure 10A), IL-6 (Figure 10B), and TNF-α (Figure 10C) were measured at 2, 12, and 24 h post-treatment. In uninfected cells, LXA4 treatment did not significantly alter the mRNA abundance of these cytokines versus the control group (CON + LXA4 vs. CON, *p* > 0.05; Figure 10).

Solitary challenge with *S. Typhimurium* (ST group) led to a marked increase in IL-1β, IL-6, and TNF-α (*p* < 0.05), confirming that *S. Typhimurium* induces a robust inflammatory response in HD11 cells. Following 2 h of LXA4 treatment in infected cells, TNF-α mRNA expression exhibited no statistically significant reduction, although a decreasing trend was observed (*p* > 0.05). Meanwhile, the mRNA transcript levels of IL-1β and IL-6 showed a marked decline during this interval (*p* < 0.05).

Relative to the ST group, LXA4 administration for 12 and 24 h (ST + LXA4 group) significantly attenuated the mRNA transcript levels of IL-1β, IL-6, and TNF-α provoked by *S. Typhimurium* challenge (*p* < 0.05). Notably, LXA4 treatment significantly suppressed the production of pro-inflammatory cytokines compared with the ST group, illustrating that LXA4 effectively attenuates the *S. Typhimurium*-induced inflammatory response in HD11 macrophages.

### 3.11. Anti-Inflammatory Mechanism of LXA4 and ALX/FPR2 Antagonist Boc-2 in S. Typhimurium-Stimulated HD11 Macrophages

**Figure 11:**
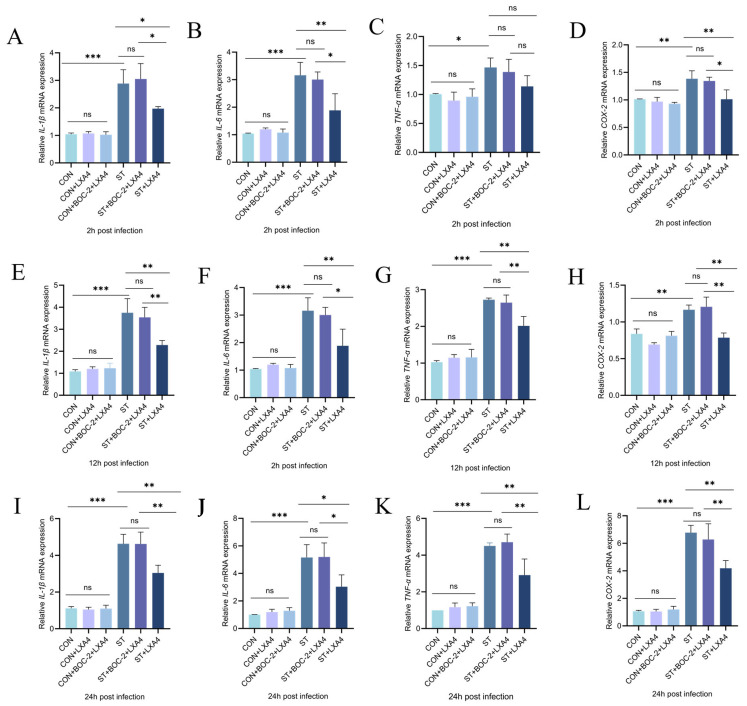
Relative mRNA expression of IL-1β (**A**,**E**,**I**), IL-6 (**B**,**F**,**J**), TNF-α (**C**,**G**,**K**), and COX-2 (**D**,**H**,**L**) in HD11 macrophages at different time points following *S. Typhimurium* infection, with or without LXA4 and the ALX/FPR2 antagonist Boc-2. ns, *p* > 0.05 (no significance); * *p* < 0.05, ** *p* < 0.01, *** *p* < 0.001 (significant).

To explore the mechanisms underlying the anti-inflammatory properties of LXA4, Boc-2, a selective antagonist of the ALX/FPR2 receptor, was utilized to disrupt receptor-mediated signaling. As shown in Figure 11, blocking the ALX/FPR2 receptor partially reversed the LXA4-induced downregulation of IL-1β, IL-6, TNF-α, and COX-2 mRNA in *S. Typhimurium*-challenged HD11 cells.

At 2, 12, and 24 h, the relative mRNA transcript levels of inflammatory mediators in normal HD11 macrophages were statistically non-significant across the control group (CON), the LXA4-treated cohort (CON + LXA4), and the group receiving the ALX/FPR2 blocker Boc-2 prior to LXA4 (CON + Boc-2 + LXA4; *p* > 0.05). These findings demonstrate that under basal conditions, neither the administration of LXA4 alone nor its combination with Boc-2 exerted any discernible influence on the mRNA transcriptional profiles of IL-1β, IL-6, TNF-α, and COX-2 in HD11 macrophages. Conversely, a robust upregulation of these pro-inflammatory mediators was observed following *S. Typhimurium* challenge, with the ST group exhibiting significantly higher mRNA transcriptional levels than the CON group (*p* < 0.05).

At 2 h, treatment of *S. Typhimurium*-stimulated HD11 macrophages with LXA4 (ST + LXA4 group) prompted a marked decline in the mRNA transcript levels of IL-1β, IL-6, and COX-2 relative to the ST group (*p* < 0.05; Figure 11A,B,D). Meanwhile, the mRNA expression level of TNF-α at this time point was statistically non-significant (*p* = 0.182) (Figure 11C). At 12 h, the ST + LXA4 treatment group displayed a pronounced decrease in the mRNA transcript levels of IL-1β, IL-6, TNF-α, and COX-2 in comparison with the ST group (*p* < 0.05; Figure 11E–H). Similarly, at 24 h, LXA4 administration substantially decreased the mRNA expression levels of IL-1β, IL-6, TNF-α, and COX-2 relative to the ST group (*p* < 0.05; Figure 11I–L).

Importantly, the suppressive effect exerted by LXA4 toward pro-inflammatory cytokine transcript levels was abolished when infected HD11 cells were simultaneously exposed to the ALX/FPR2 receptor blocker Boc-2 (ST + Boc-2 + LXA4 group) (Figure 11). Taken together, the present findings demonstrate how LXA4 mediates its anti-inflammatory actions, partially via the stimulation of the ALX/FPR2 receptor.

## 4. Discussion

*S. Typhimurium* is a significant Gram-negative enteric pathogen widely distributed among poultry, mammals, and the environment, and is a major causative agent of avian infections [28]. In poultry, infection typically induces acute intestinal inflammation, which is characterized by excessive activation of neutrophils and macrophages, resulting in intestinal barrier disruption and histopathological changes [29]. Upon necropsy, the ceca of Wenchang chicks infected with *S. Typhimurium* exhibited characteristic exudative inflammatory alterations. The cecal lumen was filled with a substantial accumulation of yellowish, viscous mucoid contents, while the cecal wall displayed marked thickening due to inflammatory edema, accompanied by diffuse mucosal congestion. These macroscopic findings are highly consistent with recent characterizations of the initial pathological features of avian salmonellosis [30,31]. Such gross lesions primarily stem from *S. Typhimurium*-induced disruption of the intestinal epithelial barrier and the subsequent induction of a robust inflammatory cascade. It is worth noting that despite the pronounced inflammatory signs, the overall intestinal architecture remained fundamentally intact, with no observable severe necrosis or hemorrhagic foci, reflecting the typical hallmark of early-stage *S. Typhimurium* infection. In contrast, the LXA4-treated group exhibited a discernible trend toward inflammatory resolution, characterized by reduced viscosity of cecal contents and mitigated congestion. This observation underscores the potential of Lipoxin LXA4 in modulating the innate immune response in poultry, likely by suppressing the overproduction of pro-inflammatory cytokines and thereby alleviating the histopathological damage triggered by *S. Typhimurium* infection [32]. The absence of histopathological scoring precludes a quantitative assessment of microscopic damage to the mucosal and submucosal layers, which may limit a comprehensive evaluation of the depth of LXA4’s protective effects.

In our study, the evaluation of inflammatory mediators was focused on the first 7 days post-hatch, capturing the acute phase of *Salmonella* infection. This period is characterized by a “cytokine storm” that often leads to high mortality in young chicks [4,33]. Our results demonstrate that LXA4 significantly suppresses the expression of IL-1β, IL-6, TNF-α, and COX-2, thereby mitigating excessive collateral tissue damage. However, it is essential to consider that the role of these mediators may shift as the infection transitions from an acute to a chronic or persistent stage [34]. Pro-inflammatory cytokines like TNF-α and IL-1β are not only drivers of acute pathology but are also critical for the activation of macrophages and the orchestration of Th1-type cell-mediated immunity, which is indispensable for the ultimate clearance of intracellular *Salmonella* [35].

As highlighted by Wigley, the avian immune response to *Salmonella* involves a delicate balance: while an overactive response causes systemic failure, an overly suppressed response may facilitate the establishment of a carrier state [36,37]. Therefore, while the LXA4-mediated reduction of these mediators is protective in the short term, its long-term impact on bacterial persistence warrants further investigation. While host immune responses are essential for pathogen clearance, an excessive release of inflammatory mediators, including IL-1β, IL-6, and TNF-α can exacerbate tissue damage [38]. While these findings are insightful, the reliance on a male-only cohort may constrain their broader generalizability [39]. Although sexual dimorphism is minimal in Wenchang chickens at this early developmental stage, future investigations incorporating larger, mixed-sex populations are warranted to further validate the robustness and universal applicability of these immune mechanisms.

As an endogenous lipid signaling molecule synthesized via the arachidonic acid (AA) metabolic pathway, Lipoxin A4 is a foundational member of the specialized pro-resolving mediator (SPM) family that orchestrates the termination of both acute and chronic inflammatory responses [40,41]. The biological actions of LXA4 are predominantly mediated via its interaction with the G protein-coupled ALX/FPR2 receptor, which is extensively distributed across neutrophils, macrophages, dendritic cells, and epithelial cells [42,43]. Boc-2 is a peptide compound that blocks the ALX/FPR2 receptor through N-terminal tert-butoxycarbonyl protection [44,45]. LXA4, via its targeted association with the ALX/FPR2 receptor, attenuates inflammatory responses by limiting neutrophil infiltration, reducing pro-inflammatory mediator production, and thereby protecting tissues from excessive damage [46,47]. The study indicates that Boc-2 prominently reverses the regulatory anti-inflammatory effects facilitated by LXA4 in *S. Typhimurium*-infected HD11 cells. Additionally, LXA4 can indirectly suppress pro-inflammatory cytokine transcription via the inhibition of the TLR4/MyD88/NF-κB signaling axis [48]; furthermore, its metabolite, 15-oxo-LXA4, has been reported to covalently modify key cysteine residues in NF-κB-associated proteins via electrophilic reactions [49].

The production of prostaglandins is primarily mediated by the inducible biocatalyst COX-2, whose expression levels are significantly elevated in response to inflammation [50]. Consistent with our observations, a marked enhancement in COX-2 mRNA expression was driven by *S. Typhimurium* stimulation in HD11 macrophages, which was efficiently abrogated by the addition of LXA4. Considering COX-2’s role in regulating local intestinal inflammation and barrier integrity [51], this inhibitory effect suggests that LXA4 contributes to limiting excessive inflammatory responses and maintaining gut homeostasis. Notably, LXA4 exhibited no direct bactericidal activity, highlighting its potential as an “immune-modulatory anti-inflammatory agent” [52,53]. This host-directed therapeutic strategy does not rely on bacterial susceptibility and may provide an alternative intervention in the context of multidrug-resistant *S. Typhimurium*, potentially complementing conventional antibiotics without increasing selective pressure for resistance.

This study employed chicken-derived HD11 macrophages as an in vitro model, which effectively recapitulated aspects of the avian intestinal immune response. However, differences between this model and the complex in vivo immune network should be considered. In mammalian research, single macrophage cultures and epithelial cell-macrophage co-culture systems (e.g., Caco-2/THP-1) have been successfully utilized to evaluate the immunomodulatory effects of prospective anti-inflammatory molecules [23,54,55,56,57]. Consequently, future studies may employ co-culture systems involving the intestinal epithelial and macrophage lineages, alongside in vivo paradigms, to further substantiate the anti-inflammatory and immunoprotective actions of LXA4.

Overall, our results demonstrate that LXA4 modulates the inflammatory milieu elicited by *S. Typhimurium* challenge in HD11 macrophages by activating the ALX/FPR2 receptor, suppressing pro-inflammatory cytokines and COX-2 expression, and enhancing cell viability under infectious conditions. As a natural lipid mediator, LXA4 shows promise as an adjunctive therapy for bacterial enteritis in poultry and provides a mechanistic and experimental foundation for non-antibiotic intervention strategies.

## 5. Conclusions

In conclusion, this study demonstrates that *S. Typhimurium* infection severely impairs intestinal health, immune homeostasis, and growth in Wenchang chickens within a controlled experimental model. LXA4 treatment showed potential in alleviating these pathological alterations in our study by suppressing the expression of inflammatory factors. Mechanistically, as a pro-resolving lipid mediator, LXA4 exerts anti-inflammatory effects by downregulating COX-2 and pro-inflammatory cytokines (IL-1β, IL-6, and TNF-α) via the ALX/FPR2 signaling pathway. Notably, within this specific experimental setting, LXA4 was observed to suppress excessive inflammation while maintaining host immune stability. These findings identify LXA4 as a potential immunomodulatory candidate for mitigating *S. Typhimurium*-induced enteritis in poultry. While these findings are primarily based on gene expression and the absence of protein-level validation is a limitation, this work provides a preliminary theoretical foundation for exploring non-antibiotic strategies. However, further extensive field trials and clinical validations are warranted to confirm its safety and efficacy in large-scale poultry production. This work provides a preliminary theoretical foundation for exploring non-antibiotic strategies to improve intestinal health and disease resistance in chickens.

## Figures and Tables

**Table 1:** Clinical Symptom Scores of *S. Typhimurium* Infection.

Score	Mental State	Fecal Characteristics
0	Active with normal rest	Normal, well-formed stool
1	Mild depression, slow response to sounds, prefers to lie down	Yellow-brown, loose stool or perianal contamination
2	Moderate depression, easily drowsy, lethargic, often motionless	Gray-white, mucous loose stool or watery diarrhea
3	Severe depression, weak, unable to stand, unresponsive to sounds	Green, loose stool or cloacal obstruction

**Table 2:** Primer sequences for qRT-PCR analysis.

Gene	Primer Sequence (5′–3′)	Size (bp)
*β-actin*	F: 5′ ACCCTGAAGTACCCCATTGAAC 3′	107
	R: 5′ TGCTCCTCACGGGCTACTCT 3′	
*TNF-α*	F: 5′ CTCAGGACAGCCTATGCCAACA 3′	177
	R: 5′ CCACCACACGACAGCCAAGT 3′	
*IL-1β*	F: 5′ AGCAGCAGCCTCAGCGAAGA 3′	183
	R: 5′ CCTCCGCAGCAGTTTGGTCAT 3′	
*IL-6*	F: 5′ AATCCCTCCTCGCCAATCT 3′	102
	R: 5′ TCACGGTCTTCTCCATAAACG 3′	
*COX-2*	F: 5′ CTGTTGGGCAGGAGGTGTTTGG 3′	126
	R: 5′ GCTGCTCATCATCCCACTCTGG 3′	

## Data Availability

The data represent the original findings of this study and are available within the article.
